# Gender differences in authorships are not associated with publication bias in an evolutionary journal

**DOI:** 10.1371/journal.pone.0201725

**Published:** 2018-08-29

**Authors:** Hannah A. Edwards, Julia Schroeder, Hannah L. Dugdale

**Affiliations:** 1 School of Biology, The Faculty of Biological Sciences, University of Leeds, Leeds, United Kingdom; 2 Department of Life Sciences, Imperial College London, Ascot, United Kingdom; Indiana University Bloomington, UNITED STATES

## Abstract

The loss of talented women from senior academic positions has partly resulted from a lower number of published papers and the accompanying reduced visibility of female compared to male scientists. The reasons for these gender-differences in authorship is unclear. One potential reason is a bias in the editorial and review process of scientific journals. We investigated whether patterns of authorship and editorial outcome were biased according to gender and geographic location in the *Journal of Evolutionary Biology*. Such potential bias may contribute to inequality in the field. We found patterns of gender differences in authorship, but this was unrelated to the editorial decision of whether to publish the manuscript. Female first-authors (the lead role) were six times less likely to be named as the corresponding author than male first-authors, and female first-authors were more likely to be displaced as corresponding authors by female co-authors than were male first-authors. We found an under-representation of female first- and last-authors compared to baseline populations of members of the European Society for Evolutionary Biology (which publishes the *Journal of Evolutionary Biology*) and of Evolutionary Biology faculty at the world top-10 universities for the Life Sciences. Also, manuscripts from Asia were five times more likely to be rejected on the final decision, independent of gender. Overall our results suggest that the peer review processes we investigated at the *Journal of Evolutionary Biology* are predominately gender-neutral, but not neutral to geographic location. Editorial gender-bias is thus unlikely to be a contributing factor to differences in authorship in this journal.

## Introduction

Across the sciences, women occupy a similar number of graduate-level positions than men but fewer senior-academic positions [1,2,3,4]. This loss of women progressing to more senior positions in scientific academia within the EU is so apparent that it is often described as the ‘leaky pipeline’, although this metaphor may fail to capture the diversity of routes through academia [4]. The collation of empirical evidence is vital to understanding the causes of this ‘leak’, so that we can work towards a more diverse and equal population of scientists in the future [5, 1, 6].

Attaining senior academic positions requires academic success, which is to a large degree demonstrated by publishing many, widely and positively cited pieces of research in highly influential journals [5]. A reduced publication record and the associated low visibility within a research field impacts negatively on the careers of scientists [7]. Yet, in the field of Ecology and Evolution, women publish 40% less than men [8] and across multiple disciplines in science, women account for less than 30% of fractionalised authorships [9]. Furthermore, women are underrepresented as first and last authors, which in the field of Ecology and Evolution represent the most prestigious lead and senior authorship positions, respectively [10]. Women are also overrepresented as middle authors in Ecology, and Cell and Molecular Biology [10,11]; middle authorship is considered the least prestigious authorship position. These gender differences contribute to the low visibility of women scientists in these fields. For example, the work of female scientists is represented two to three times less than expected in Ecology text books [7] and women are under-represented as invited speakers at Evolutionary Biology conferences [12]. Such low visibility leads to fewer female role models to inspire future generations of scientists, which only perpetuates the problem.

What causes gender patterns in authorship remains unclear. It may be that women are perceived to have more junior roles in scientific research projects, or that women are less likely to self-promote [13] and have a lower perception of their success [14,15], and so may fail to negotiate authorship position successfully. Men and women may differ in their approach to, and the success of, negotiations for authorship positions. Additionally, there may be a gender bias in the editorial and review process of scientific journals [16]. Such implicit (unconscious) bias acts in both men and/or women [17].

To better understand the causes of the ‘leaky pipeline’ and gender patterns in authorship, journals can quantitatively review their editorial and review process. For example, it has been suggested that the removal of author and reviewer identity, known as the double blind review, may reduce gender bias [18]. However, other studies have not found support for this notion [19,20]. Author gender may thus be more of a perceived rather than a contributing factor to inequality in publishing [21,22]. Furthermore, women are underrepresented as the decision-makers on the editorial boards of scientific journals, with disproportionately more men serving on editorial boards than women [23,24,25,26], although certain journals are trying to redress this imbalance [27]. This underrepresentation could influence reviewer selection due to differences in the criteria selection of reviewers by female versus male editors [28,29], or the professional networks of female versus male editors [30]. Editor gender may also influence the decisions of reviewers to accept to review a manuscript [31]. These factors may ultimately affect the outcome of the publication process.

An underrepresentation of geographic location may also influence patterns of authorship. There is variation by geographic locations of members on the editorial boards of scientific journals, with scientists from the United States dominating editorial boards [32,33,34]. Similar to the underrepresentation of women on editorial boards, underrepresentation of geographic location may influence aspects of the peer review process. This is because professional networks and affiliations can affect the outcome of a manuscript [22], author location can influence manuscript publication [35] and citation rate [36], and reviewer location can bias the decision to review and the review outcome [31].

In this study, we used the database of the reviewing requests and publication decisions of manuscripts submitted to the European Society for Evolutionary Biology’s journal, the *Journal of Evolutionary Biology* (*JEB*), between 2012–2016 to test several hypotheses: (1) Women are underrepresented as first and last authors of scientific papers [9], which represent the most prestigious lead and senior authorship positions in Evolutionary Biology, and on editorial boards [23,24,25,26] and as reviewers [31]. We therefore expect that there is a difference in representation between the genders of first authors, last authors, editors and reviewers at the *JEB*. (2) The loss of women from senior academic positions [1,2,3,4], may result in their corresponding authorship position being deferred to their graduate or post-doctoral advisors. We therefore expect that the gender of the first author is associated with whether the first author is a corresponding author, a status generally assigned to first or last authors. (3) As scientists are more likely to collaborate on papers with researchers of the same gender [37, 16, 38], we expect that the gender of the first author is associated with the gender of the last author. (4) Gender patterns in authorship can differ by geographic location [9], we expect that the gender of the first author may be associated with the first author’s continent of affiliation. (5) Since author gender [18] and affiliation [22] may influence peer-review outcomes, we expect the handling editor’s decision to send a manuscript for review, the reviewer’s decision to review a manuscript, and, the first and final publication decisions on a manuscript to be associated with the gender of the first author, the gender of the last author, the first author’s continent of affiliation, and the gender of the reviewer.

## Materials and methods

### Dataset and assigning gender

The editorial office of the *JEB*, the society journal for the *European Society for Evolutionary Biology* (*ESEB*), provided data on the reviewing requests and publication decisions of manuscripts submitted to the journal between January 2012 and February 2016. The names of authors were not blinded from reviewers during this period. All manuscript types (research paper, reviews, short notes, special issue) were included in the analyses. The *JEB* assigned a handling editor to 3,348 unique manuscripts: 2,722 (81%) were sent for review with 19% being rejected. Of these 3,348 manuscripts, 1,814 (54%) were invited for revision, 1,424 (43%) were rejected, 30 (0.01%) were accepted and 80 had an unknown decision (0.02%). 778 manuscripts with revisions required were resubmitted and 677 (87%) of those were accepted. The ESEB office also provided membership data between 2012 and 2016 to generate baseline gender data.

For individuals that had an initial as a first name we performed an internet search (using their initial, surname and country of affiliation on google.co.uk; N = 35) and searched for sources that included a photograph of the individual, such as individual web pages or online profiles that allowed us to assign a first name (N = 27). Gender was then assigned to first names using genderizeR 2.0.0 [39] in R 1.1.419 [40]. The package genderizeR uses the online database genderize.io, which includes >200,000 unique names. For each first name provided, genderize.io returns a count of the number of times the name appears in the database, and a corresponding gender probability based on frequency counts. To increase the accuracy of the gender predictions we followed the method of Topaz and Zen [41], and calculated a modified probability, using the probability *p* and count *c* as *P*_*mod*_ = *pc*+2/*c*+4, and then included gender *P*_*mod*_ ≥ 0.85. Gender was assigned to: all editors, 79% of first authors, 80% of last authors, 79% of corresponding authors and 84% of reviewers.

We validated the gender assigned to names that had a *P*_*mod*_ between 0.85–0.855 by searching (using their first name, surname and country of affiliation) online sources that included a photograph of the individual, such as individual web pages or online profiles. All of the five names within this probability range had genders that agreed with our online searches. We also took 30 random male and 30 random female names assigned a gender and performed online searches, similar to our previous searches, to validate their gender assignment. We could not validate 8 names by an individual web page or online profile, so we performed a google image search to identify the gender normally associated with these eight names. All of the 60 names within this subsample had assigned genders that agreed with our online searches.

### Statistical analyses

All statistical analyses were performed in R 1.1.419 [40] using Generalised Linear Mixed Models (GLMM) in MCMCglmm 2.17 [42]. We specified V = 1 and n = 2 for the residual, an Inverse Wishart structure for the random effects and used a binomial distribution with log link for all models. We sampled the posterior distribution every 250 iterations, with a burn-in period of 10,000 iterations and a run of 403,000 iterations. We assessed convergence using the heidel.diag and geweke.diag functions, and inspected the autocorrelation values (*r* < 0.1) and time series plots. Beta coefficients were interpreted using the “divide by four” rule [43], and pMCMC values were corrected for false discovery rate (FDR) [44].

### (1) Author gender distribution

We first described the data and tested whether the first-author’s gender (here and in all following instances: male/female) was associated with the last-author’s gender, the gender of the corresponding author, the first-author’s continent of affliation (Africa, America, Asia, Europe, Oceania) and whether the first author was the corresponding author (yes/no). We also included year of submission date and manuscript type (research papers/reviews/short notes/special issue) as fixed effects, and manuscript number as a random effect (to account for resubmitted manuscripts). To assess whether the gender of the corresponding author was independent of first-author gender, for first authors who were not corresponding authors, we compared the counts of males and females who were first authors but not corresponding authors, to the counts of males and females who were the corresponding author using a chi-squared test.

#### Membership gender comparison

We used the rbinom function in R to compare the gender ratio of first authors, last authors, reviewers and editors, to the gender ratio from 10,000 randomisations of the data. The randomisations allow for the comparison of the mean annual gender ratios from our baseline populations. These were taken from: 1) the members of ESEB between 2012–2016, which we expect to contribute to first-author papers, 2) the academic faculty (Fellows, Lecturers and Professors in 2013, [12]) belonging to the Evolutionary Biology departments at the world top-10 universities for the Life Sciences, which we expect to mostly represent last-authors. The universities were selected using the Times Higher Education University Ranking 2010–2011 and included Evolutionary Biology departments at: Imperial College London, MIT, Harvard University, Princeton University, Stanford, University of California Berkeley, University of Cambridge, University College London, University of Oxford, University of Yale. 3) the senior (non-student) members of ESEB between 2012–2016, which we expect to represent reviewers and editors. In each randomisation, an individual was randomly selected 3,348 times (the number of unique manuscripts assigned a handling editor by the *JEB*) for the first author/last author/reviewer simulation and 93 times (the number of unique handling editors at the *JEB*) for the editor simulation and assigned a gender, based on the gender ratios of our baseline populations.

### (2) Handling editor

We then tested for an association of author and handling-editor gender and whether the manuscript was sent for review (yes/no), regardless of the manuscript’s final outcome, and agreed to be reviewed (yes/no). The model contained the first-author’s gender and handling-editor’s gender, an interaction between the two, and also the last-author’s gender, the first author’s continent of affiliation (Africa, America, Asia, Europe, Oceania), year and manuscript type (research papers/reviews/short notes/special issue) as fixed effects. We also included editor identity and manuscript number as random effects to account for re-occuring editors and manuscripts.

### (3) Reviewer

We then tested for an association between the gender of both author and handling-editor, and whether the reviewer agreed to review the manuscript (accept/decline). The model contained the first-author’s gender and an interaction of it with the handling-editor’s gender, the reviewer’s gender and an interaction of the reviewer’s gender with the handling-editor’s gender. We also included the last-author’s gender, the first-author’s continent of affiliation (Africa, America, Asia, Europe, Oceania), year and manuscript type (research papers/reviews/short notes/special issue) as fixed effects. We also included editor and reviewer identitiy and manuscript number as random effects to account for re-occuring editors, reviewers and manuscripts.

### (4) Editorial outcome

We finally tested for the effect of author and reviewer gender on the outcome of the journal’s: 1) first decision (accept/revise or reject) and, 2) final decision (accept/reject). The model contained the first-author’s gender and an interaction with the reviewer’s gender, the last-author’s gender, the first-author’s continent of affiliation (Africa, America, Asia, Europe, Oceania), year and manuscript type (research papers/reviews/short notes/special issue) as fixed effects. We also included reviewer and manuscript identity as random effects to account for re-occuring reviewers and manuscripts.

## Results

### (1) Author gender distribution

**Fig 1:**
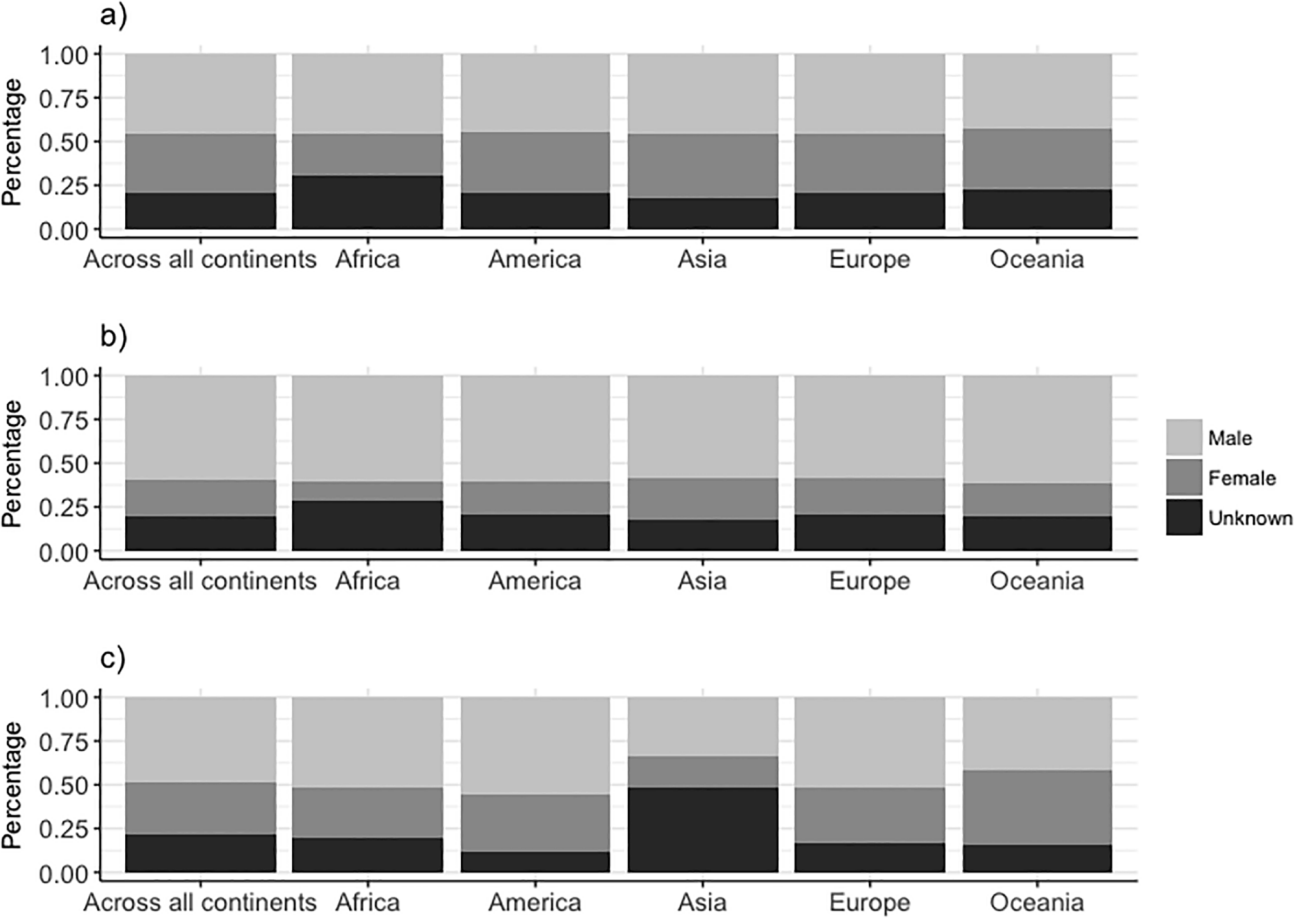
The percentage of men and women as a) first, b) last and, c) corresponding authors, and broken down by continent of affiliation. This is based on the 3,348 manuscripts (N: Africa = 46, America = 1051, Asia = 385, Europe = 1664, Oceania = 202) submitted to the *JEB* between 2012–2016, and the percentages of authors with unassigned gender are also shown.

**Fig 2:**
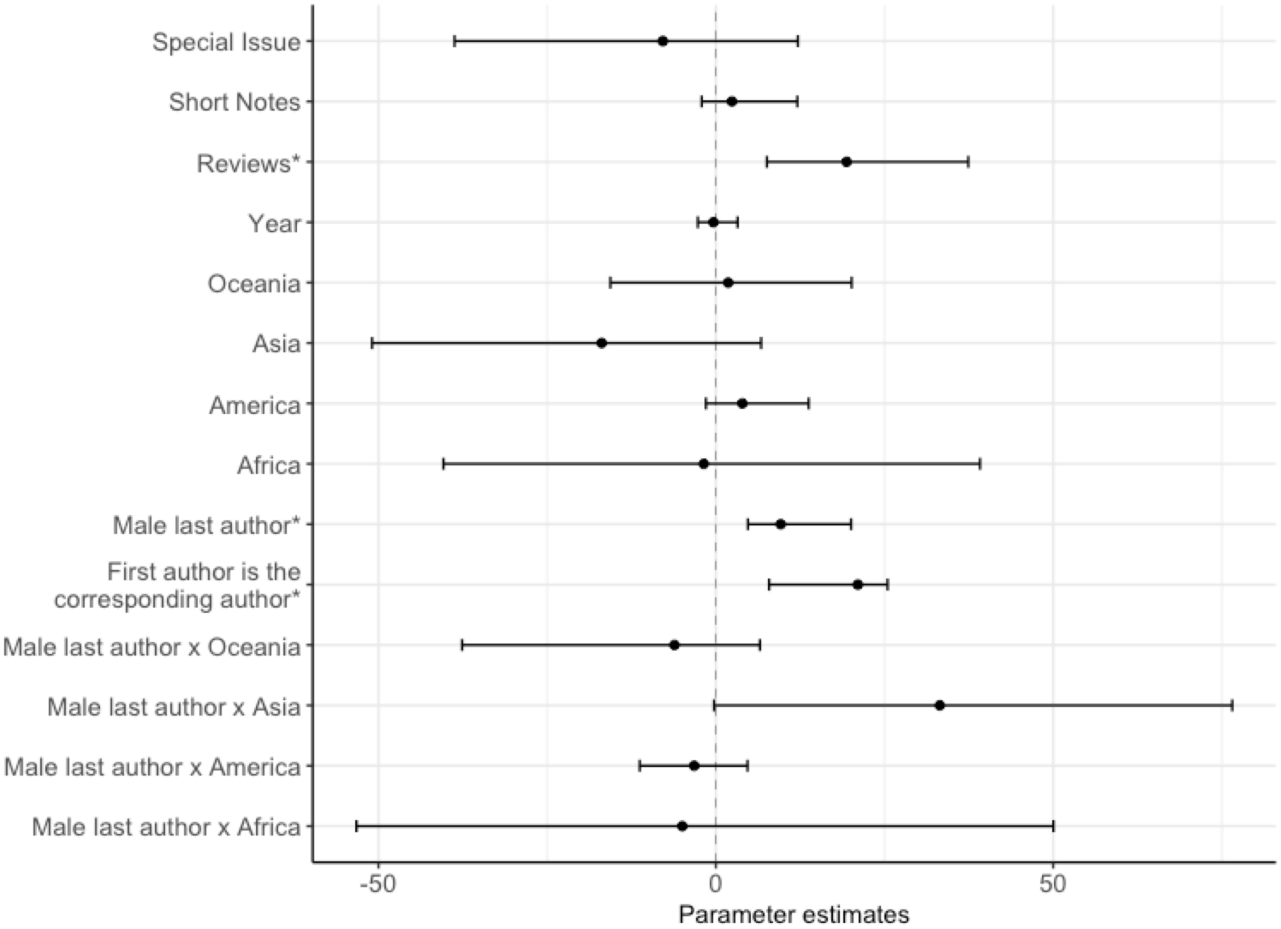
Factors predicting the gender of the first author, showing the posterior mode estimates for the fixed effects in the binomial model. The model contained: manuscript type (N: research paper = 1,916, reviews = 88, short notes = 243, special issue = 17; contrast level = research paper), year, continent of affiliation (N: Africa = 26, America = 795, Asia = 82, Europe = 1,208, Oceania = 153; contrast level = Europe), gender of the last author (N: male = 1,697, female = 567; contrast level = female), whether the first author is the corresponding author (N: first author is the corresponding author = 1,818, first author is not the corresponding author = 446; contrast level = first author is not the corresponding author), and the interaction between the gender of the last author and the continent of affiliation. An asterix (*) indicates posterior modes whose 95% credible intervals do not overlap zero, after FDR correction.

**Fig 3:**
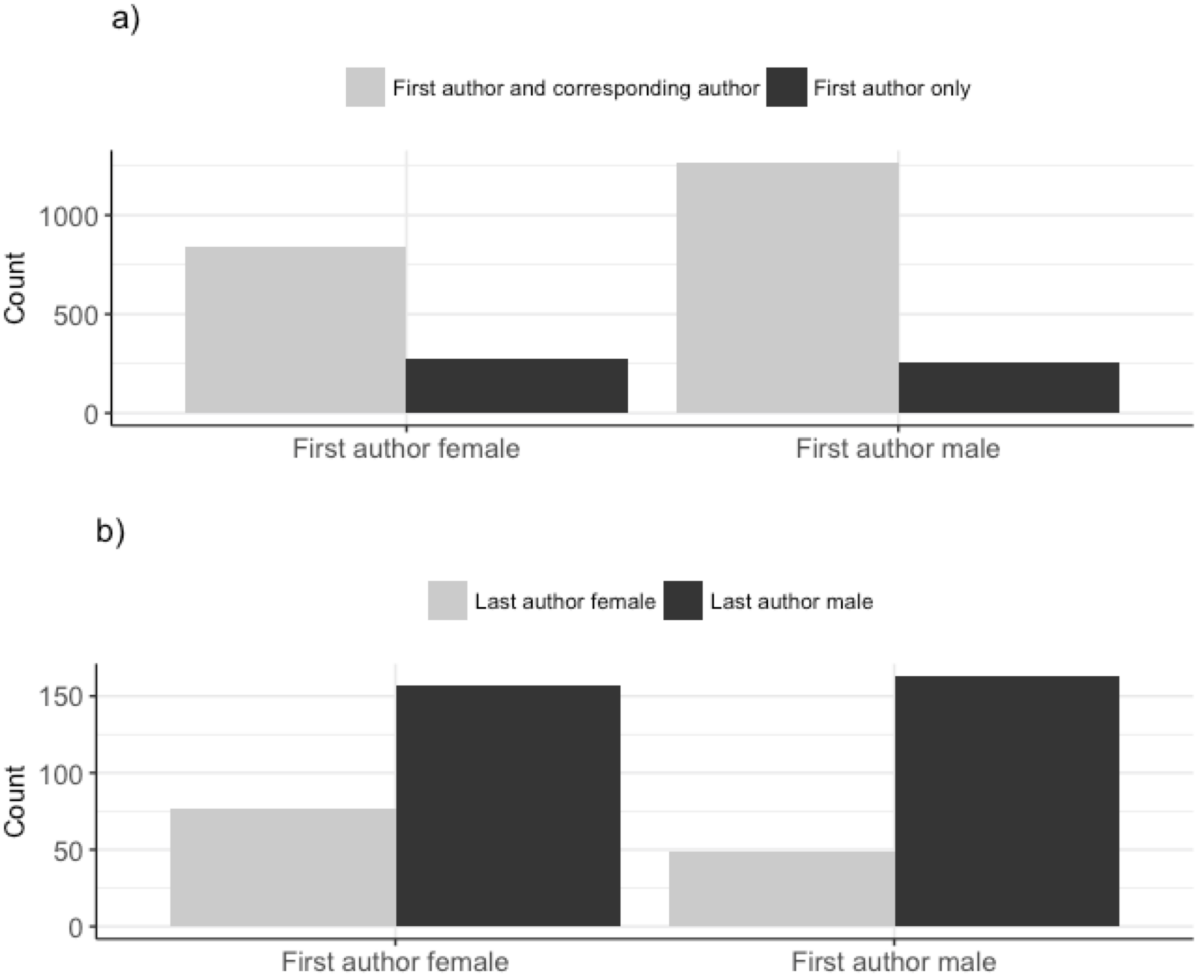
**Fig 3a. The number of male and female first authors who were or were not the corresponding author**. N: female first authors that were corresponding authors = 839, male first authors that were corresponding authors = 1,265, female first authors that were not corresponding authors = 275, and male first authors that were not corresponding authors = 258. The total number of manuscripts does not total 3,348 (the number unique manuscripts assigned a handling editor by the *JEB*) due to authors with unassigned genders. **Fig 3b. The number of male and female first authors who were not the corresponding author and the gender of the corresponding author**. N: female first authors displaced by female last author = 77, female first authors displaced by male last author = 157, male first authors displaced by female last author = 49, male first authors displaced by male last author = 163). The total number of manuscripts does not total 3,348 (the number unique manuscripts assigned a handling editor by the *JEB*) due to authors with unassigned genders.

**Fig 4:**
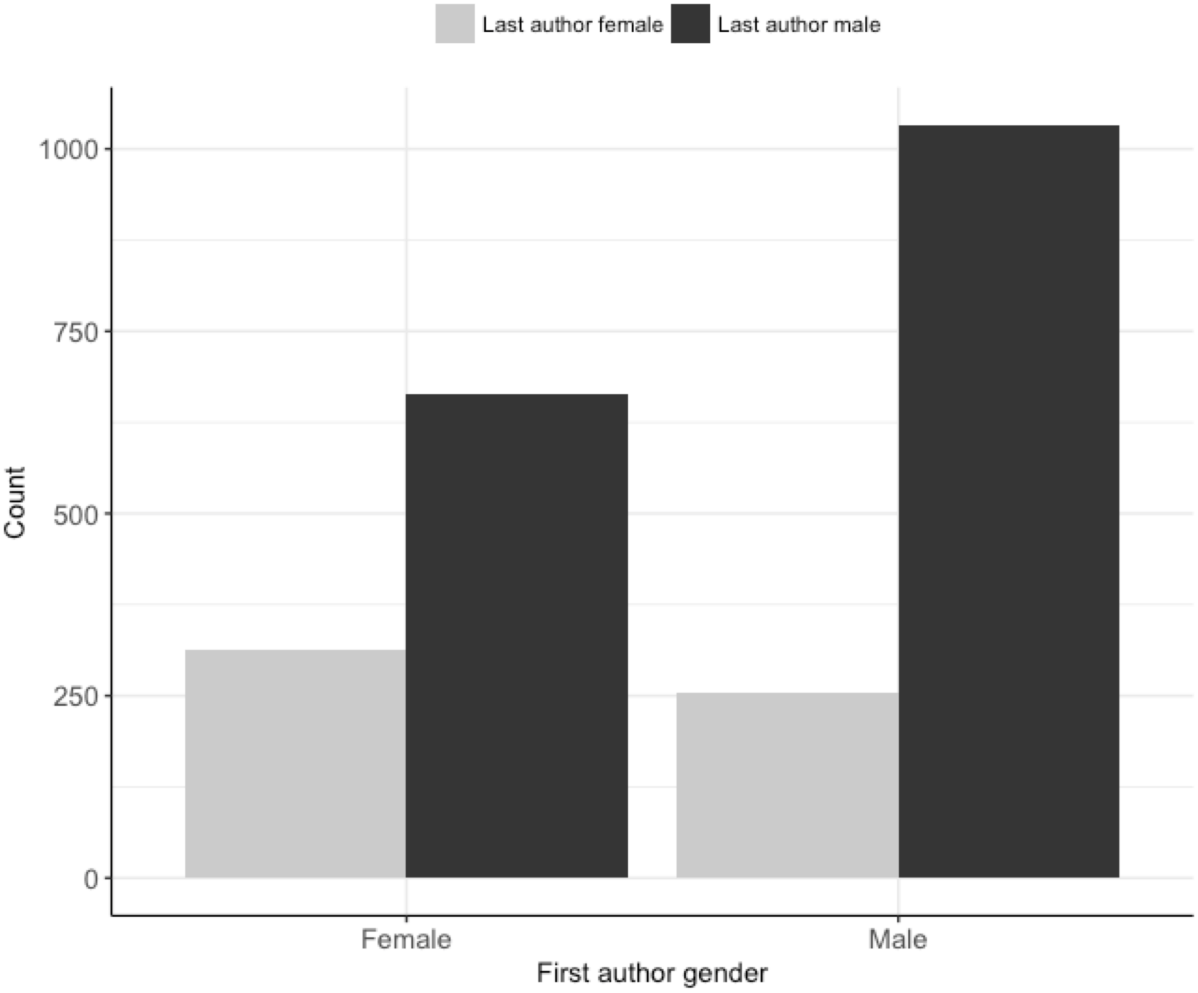
The number manuscripts with male and female first authors and the gender of the corresponding last author. N: female first author and female last author = 312, female first author and male last author = 663, male first author and female last author = 255, and male first author and male last author = 1033. Manuscript number does not total 3,348 manuscripts (the number unique manuscripts assigned a handling editor by the *JEB*) due to authors with unassigned genders.

On manuscripts submitted to the *JEB*, there were fewer women as first (42%), last (25%) and corresponding authors (37%) compared to men (Fig 1). The propensity for first authors to be the corresponding author differed between the genders, with first-author males six times more likely to be named as the corresponding author than first-author females (Table A in S1 File, Figs 2 & 3a). The gender of the corresponding author was associated with the gender of the first author (χ^2^ = 7.45, d.f. = 1, p = 0.01). When the first-author was female, they were more likely to be displaced as corresponding author by a female co-author as the corresponding author than when the first-author was male (Fig 3b). Male last authorships were three times more likely when the first author was also male compared to when the first author was female (Table A in S1 File & Fig 4). Review type papers were also more likely to be first-authored by males than females (Table A in S1 File & Fig 2). Continent of affiliation was not associated with the first author’s gender (Table A in S1 File and Figs 1 & 2).

#### Membership gender comparison

**Fig 5:**
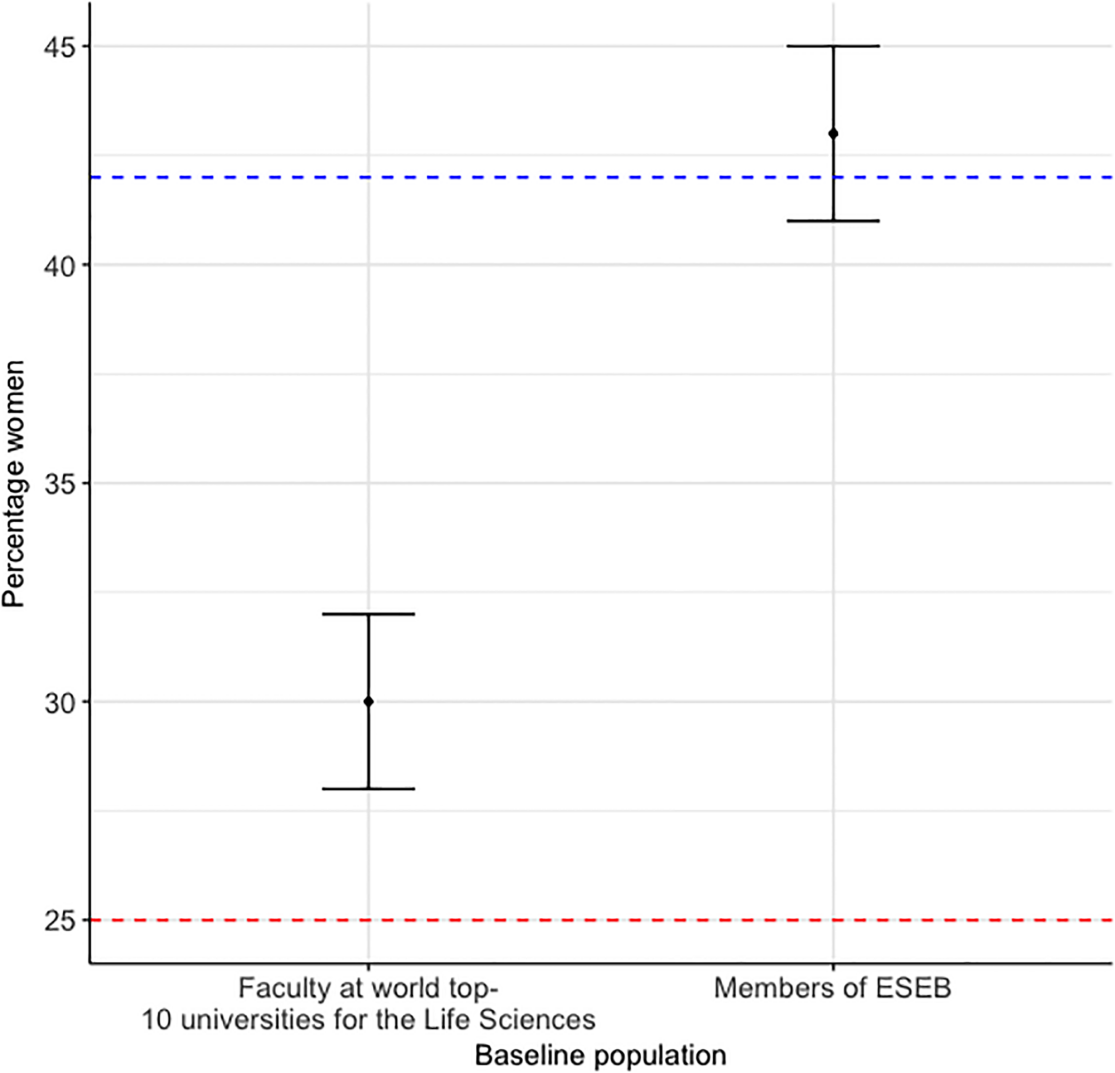
The percentage of women selected by randomizations from baseline populations of ESEB members and faculty members. Error bars = 95% confidence intervals. The blue horizontal dashed line represents the percentage of first authors (42%) that were women and the red horizontal dashed line percentage of last authors (25%) that were women on manuscripts sent to the *JEB* between 2012–2016.

The 42% of female first-authors did not differ statistically from the predicted 43% (95% confidence interval: 41–45%) from the baseline population of members of ESEB, assuming both genders had the same probability to publish. The 25% of female last-authors was lower than the predicted 30% (95% confidence interval: 28–32%) from the baseline population of academic faculty belonging to the Evolutionary Biology departments at the world top-10 universities for the Life Sciences (Fig 5). The 22% of female reviewers was lower than the predicted 38% (95% confidence interval: 36–39%) from the baseline population of senior members of ESEB. The 32% of female editors was not significantly different than the predicted 38% (95% confidence interval: 28–47%), from the baseline population of senior members of ESEB.

### (2) Handling editor

**Fig 6:**
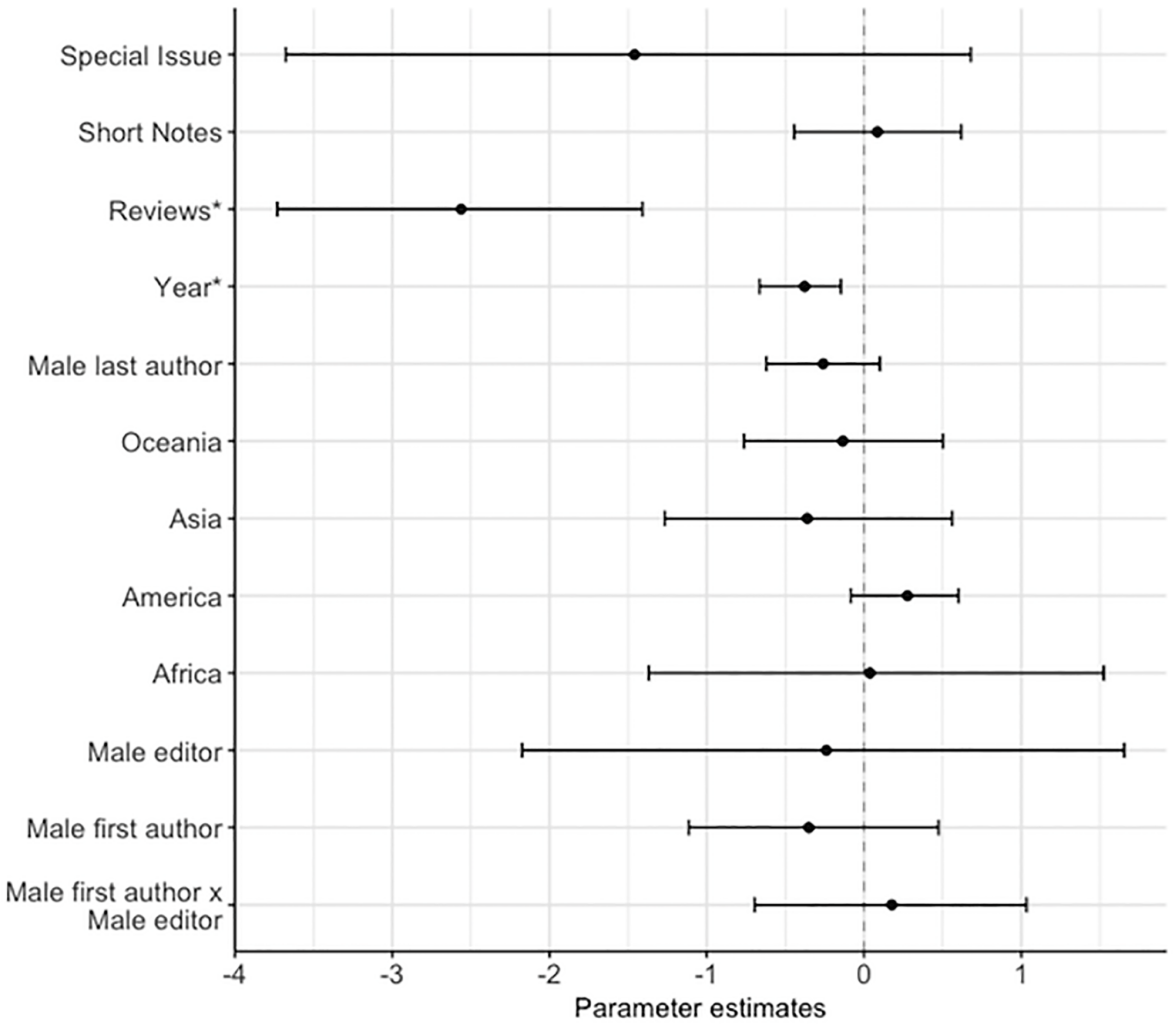
Factors predicting whether the manuscript was sent out for review, showing the posterior mode estimates for the fixed effects in the binomial model. The model contained: manuscript type (N: research paper = 1,914, reviews = 88, short notes = 242, special issue = 15; contrast level = research paper), year, gender of the last author (N: male = 1,693, female = 566; contrast level = female), continent of affiliation (N: Africa = 26, America = 794, Asia = 81, Europe = 1,205, Oceania = 153; contrast level = Europe), gender of the editor (N: male = 1,920, female = 339; contrast level = female), gender of the first author (N: male = 1,286, female = 973; contrast level = female), and the interaction between the gender of the first author and the editor. * indicates posterior modes whose 95% credible intervals do not overlap zero, after FDR correction.

The first author’s gender, last author’s gender, and editor’s gender were not associated with whether the manuscript was sent for review (Table B in S1 File & Fig 6). Independent from gender, reviews were less likely to be sent for review, regardless of the outcome, than research papers (Table B in S1 File & Fig 6). Also, manuscripts were less likley to be sent for review in more recent years (Table B in S1 File & Fig 6).

### (3) Reviewer

The reviewer’s gender, first author’s gender, last author’s gender and the editor’s gender were not associated with whether the reviewer agreed to review the manuscript or not (Table C in S1 File).

### (4) Editorial outcome

**Fig 7:**
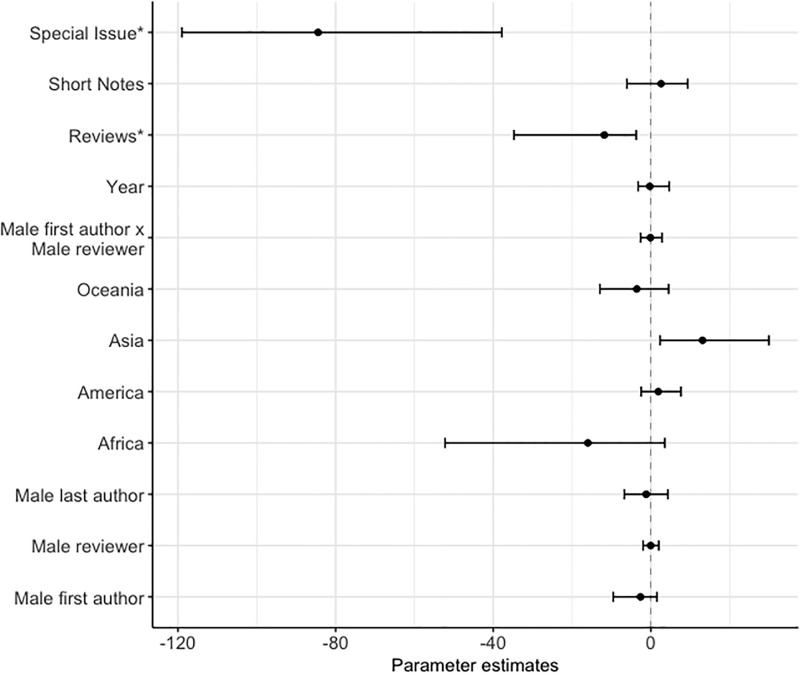
Factors predicting the first decision (accept/revise or reject) on the manuscript’s outcome, showing the posterior mode estimates for the fixed effects in the binomial model. The model contained: manuscript type (N: research paper = 1689, reviews = 52, short notes = 200, special issue = 15, contrast level = research paper), year, interaction between the gender of first author and gender of the reviewer, continent of affiliation (N: Africa = 19, America = 683, Asia = 51, Europe = 1066, Oceania = 137, contrast level = Europe), gender of the last author (N: male = 1463, female = 493, contrast level = female), gender of the reviewer (N: male = 1509, female = 447, contrast level = female) and gender of the first author (N: male = 1105, female = 851, contrast level = female). An asterix (*) indicates posterior modes whose 95% credible intervals do not overlap zero, after FDR correction.

**Fig 8:**
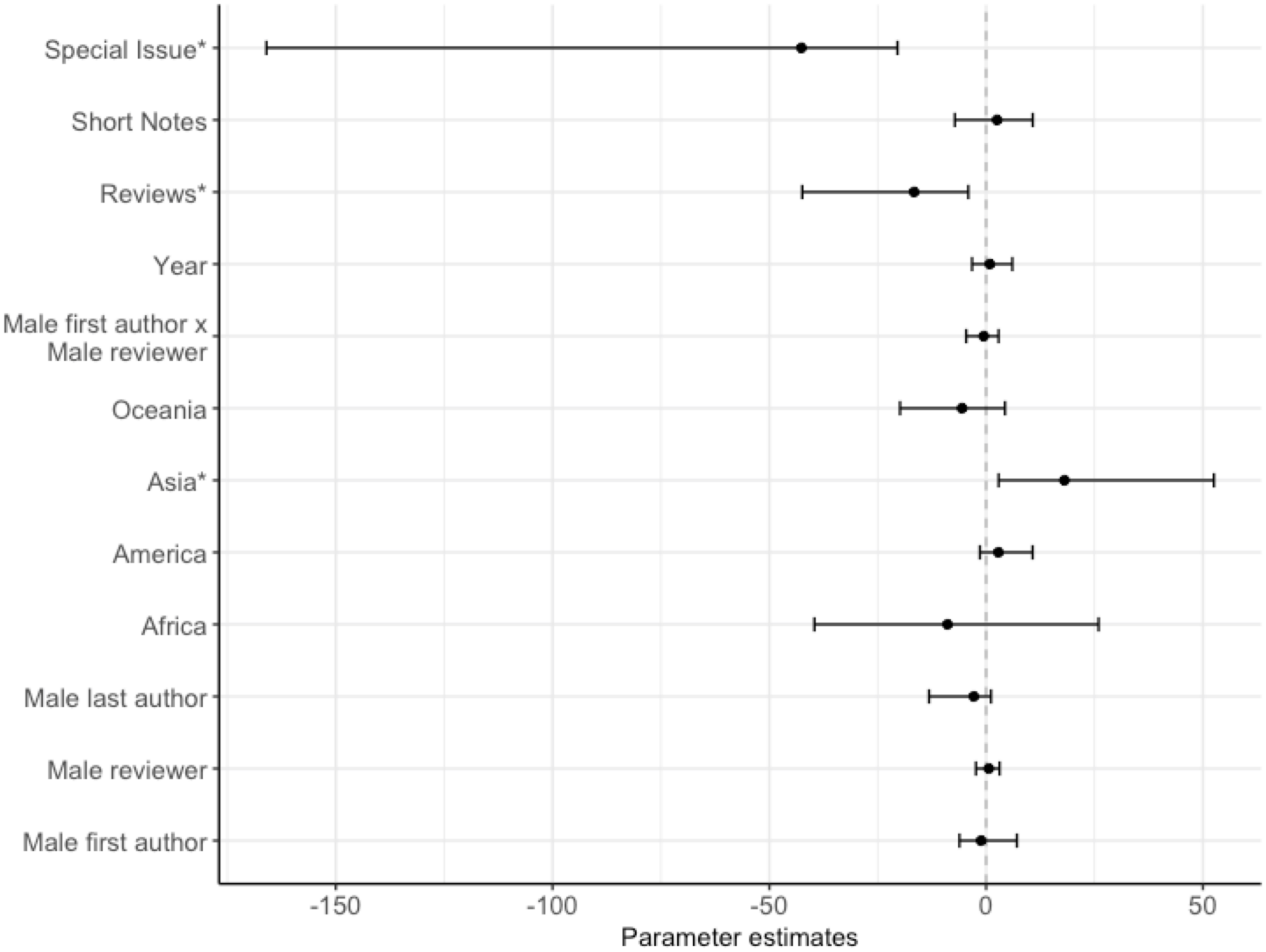
Factors predicting the final decision (accept or reject) on the manuscript’s outcome, showing the posterior mode estimates for the fixed effects in the binomial model. The model contained: manuscript type (N: research paper = 1689, reviews = 52, short notes = 200, special issue = 15, contrast level = research paper), year, interaction between the gender of first author and gender of the reviewer, continent of affiliation (N: Africa = 19, America = 683, Asia = 51, Europe = 1066, Oceania = 137, contrast level = Europe), gender of the last author (N: male = 1463, female = 493, contrast level = female), gender of the reviewer (N: male = 1509, female = 447, contrast level = female) and gender of the first author (N: male = 1105, female = 851, contrast level = female). An asterix (*) indicates posterior modes whose 95% credible intervals do not overlap zero, after FDR correction.

The first author’s gender, reviewer’s gender, and last author’s gender were not associated with the mansucript’s first editorial outcome (Table D in S1 File & Fig 7). The first author’s gender, reviewer’s gender, last author’s gender and continent of affiliation were not associated with the mansucript’s final outcome (Table E in S1 File & Fig 8). Manuscripts from Asia were five times more more likely to be rejected on the final decision than manuscripts from the contrast contintent of Europe. Reviews and special issues were also more likely to be revised or accepted on the first and final decision than research papers (Tables D-E in S1 File & Figs 7 and 8).

## Discussion

Scholarly publishing can act as an indicator and potential contributor to gender inequality in the sciences. In this study we have highlighted gender patterns of authorship in the *JEB*, but gender was not a contributing factor in the editorial decision to publish a manuscript.

Female first-authors were less likely to be named as the corresponding author than male first-authors, and this was unaffected by the gender of the last author. Female first-authors were also less likely to serve as corresponding authors than men in the journals *Functional Ecology* [16] and *Central Journal of Medicine* [45]. Fox *et al*. [16] proposed that this discrepancy may be due to women leaving science at a higher rate than do men and deferring their corresponding authorship position to their graduate or post-doctoral advisors. It may also be that women are less likely to self-promote than men [13] and/or have a lower perception of their success [14,15], and therefore may be more likely to fail to negotiate authorship position successfully.

We found female first-authors were more likely to be displaced as corresponding authors by female rather than male co-authors, compared to displaced male first-authors. Also, male last-authors were more prevalent on manuscripts with male first-authors. Several studies have found that men and women are more likely to collaborate on papers with researchers of the same gender [36, 16, 45], and this could explain our result. Gender differences in research fields and interests [46, 18, 45], and the perceived importance by women of having a mentor of the same gender [47], are potential reasons for why this non-random association between gender and co-authors may occur.

The discrepancy in senior roles between men and women was also highlighted when fewer women were last-authors than expected when compared with the gender ratio of faculty at the world top-10 universities for the Life Sciences, and also, fewer women were reviewers than expected compared with the gender ratio of ESEB senior members. The low number of female authors may be explained by the lower publication rate of women, compared to men. This effect has been noted across scientific disciplines e.g. [48], including the fields of both Ecology and Evolutionary Biology [8]. This discrepancy in publication output between men and women, however, is unknown, and often termed the ‘productivity puzzle’ e.g. [48]. Potential explanations include a culmination of reduced success in grant rounds [8], a greater proportion of time dedicated to childcare [49], a greater involvement with non-research responsibilities to balance gender on administrative committees [50], and the lower ranking of women in science [1,2,3,4], compared to men.

There are some limitations in regards to the gender analyses we wish to note. Firstly, gender was not associated with the editorial decision on a manuscript. Although this may suggest that the peer-review process at the *JEB* is predominately gender neutral, it is worth noting, that there may be gender biases we have not tested for. For example, the gender ratio for reviewers in our dataset was male biased and this may indicate that men are more likely to be suggested by authors or selected by editors as reviewers than women. Secondly, gender was not assigned to 21% of first authors, 20% of last authors, 21% of corresponding authors, and 16% of reviewers (all editors were assigned gender), and this may have limited our ability to detect certain relationships.

Finally, continent of affiliation was associated with the final editorial decision; manuscripts sent from Asia were more likely to be rejected than those from Europe. One of the main reasons for rejection is manuscripts do not fit the scope of the journal e.g. [51]. This mismatch of manuscript and journal might be more prevalent for manuscripts sent from Asia, considering there is a strong emphasis on research output in this region [52] which may encourage submissions to journals outside of the research’s scope. Language and writing styles are highlighted as problems for manuscripts written by non-native speakers [53] and may cause journals to reject manuscripts. The language and writing skills barrier may therefore be influencing the decision to reject a manuscript sent from Asia. This relationship was not seen for manuscripts sent from Africa where non-native speaker barriers may similarly apply, although the sample size from Africa was small (N = 19, versus N = 51 from Asia).

## Summary

We found gender differences in patterns of authorship, but this did not result in publication bias at the *JEB*. However, female first-authors were six times less likely to be named as the corresponding author than male first-authors. Additionally, the percentage of female first- and last-authors on manuscripts submitted to the *JEB* was significantly lower than the baseline population of ESEB members and faculty at the world top-10 universities for the life sciences. These results highlight the subtle ways gender disparity continues to exist. Manuscripts from Asia were also five times more likely to be rejected on the final decision, independent of gender. Overall, the peer review processes we investigated at the *JEB* are predominately gender-neutral, but not neutral to geographic location. Editorial gender bias is thus unlikely to be a contributing factor to differences in authorship in this journal.

## Ethical statement

The *JEB* collated the data and provided the dataset for us to analyse. The University of Leeds ethical review committee confirmed that ethical approval was not required and waived the requirement for participant consent. The *JEB* editor-in-chief and the ESEB office approved the study and publication of the final datasets. The anonymised data underlying the findings described in the manuscript are fully available without restriction at the figshare repository: https://doi.org/10.6084/m9.figshare.5821110.v1.

## Supporting information

S1 File Contains Table A: Factors predicting the gender of the first author, showing the posterior mode estimates for the fixed effects in the binomial model; Table B: Factors predicting whether the manuscript was sent out for review, showing the posterior mode estimates for the fixed effects in the binomial model; Table C: Factors predicting whether the reviewer agreed to review the manuscript, showing the posterior mode estimates for the fixed effects in the binomial model; Table D: Factors predicting the first decision (accept/revise or reject) on the manuscript’s outcome, showing the posterior mode estimates for the fixed effects in the binomial model; Table E: Factors predicting the final decision (accept or reject) on the manuscript’s outcome, showing the posterior mode estimates for the fixed effects in the binomial model.
(DOCX)
