# Correction: Gender differences in authorships are not associated with publication bias in an evolutionary journal

**DOI:** 10.1371/journal.pone.0217251

**Published:** 2019-05-22

**Authors:** Hannah A. Edwards, Julia Schroeder, Hannah L. Dugdale

In the article byline, Julia Schroeder should be listed as co-corresponding author for this paper. Dr. Schroeder’s email address is julia.schroeder@imperial.ac.uk.
