# Supplementary material for: Gender differences in authorships are not associated with publication bias in an evolutionary journal
Source: PLoS One. 2018 Aug 29;13(8):e0201725. doi: 10.1371/journal.pone.0201725 (PMC6114708; doi:10.1371/journal.pone.0201725)
Supplement: S1 File — Contains Table A: Factors predicting the gender of the first author, showing the posterior mode estimates for the fixed effects in the binomial model; Table B: Factors predicting whether the manuscript was sent out for review, showing the posterior mode estimates for the fixed effects in the binomial model; Table C: Factors predicting whether the reviewer agreed to review the manuscript, showing the posterior mode estimates for the fixed effects in the binomial model; Table D: Factors predicting the first decision (accept/revise or reject) on the manuscript’s outcome, showing the posterior mode estimates for the fixed effects in the binomial model; Table E: Factors predicting the final decision (accept or reject) on the manuscript’s outcome, showing the posterior mode estimates for the fixed effects in the binomial model. (DOCX) [file pone.0201725.s001.docx]

**Supporting material**

**Gender differences in patterns of authorship does not cause publication bias in an evolutionary journal**

**Supplementary Table A:** Table of the factors predicting the gender of the first author

**Supplementary Table B:** Table of the factors predicting whether a manuscript was sent for review

**Supplementary Table C:** Table of the factors predicting whether a manuscript was agreed to be reviewed

**Supplementary Table D:** Table of the factors predicting a manuscript’s first decision

**Supplementary Table E:** Table of the factors predicting a manuscript’s final decision

**Table A**: Factors predicting the gender of the first author, showing the posterior mode estimates for the fixed effects in the binomial model containing: whether the first author is the corresponding author (N: first author is the corresponding author = 1818, first author is not the corresponding author = 446; contrast level = first author is not the corresponding author), the gender of the last author (N: male = 1697, female = 567; contrast level = female), the continent of affiliation (N: Africa = 26, America = 795, Asia = 82, Europe = 1208, Oceania = 153; contrast level = Europe), the manuscript type (N: research paper = 1916, reviews = 88, short notes = 243, special issue = 17; contrast level = research paper), year, and the interaction between the gender of the last author and the continent of affiliation. Bold text indicates posterior modes whose 95% credible intervals do not overlap zero, after FDR correction.

|  | Posterior mode estimate | Lower credible interval | Upper credible interval | pMCMC | FDR pMCMC |
| --- | --- | --- | --- | --- | --- |
| **First author is the  corresponding author** | **21.0520** | **7.8915** | **25.4602** | **<6e-04** | **<6e-04** |
| **Male last author** | **9.6210** | **4.7553** | **20.0488** | **<6e-04** | **0.0005** |
| Africa | -1.7865 | -40.3710 | 39.1583 | 0.9288 |  |
| America | 3.9286 | -1.4658 | 13.7577 | 0.1730 |  |
| Asia | -16.9152 | -50.9519 | 6.7126 | 0.1921 |  |
| Oceania | 1.8308 | -15.6297 | 20.1328 | 0.9720 |  |
| Year | -0.3565 | -2.6712 | 3.2242 | 0.9466 |  |
| **Reviews** | **19.3853** | **7.5699** | **37.4157** | **<6e-04** | **0.0003** |
| Short Notes | 2.3870 | -2.0913 | 12.0830 | 0.2099 |  |
| Special Issue | -7.8591 | -38.7298 | 12.1615 | 0.3384 |  |
| Male last author x Africa | -4.9701 | -53.2649 | 50.0381 | 0.7990 |  |
| Male last author x America | -3.2139 | -11.2807 | 4.6978 | 0.3359 |  |
| Male last author x Asia | 33.1926 | -0.2637 | 76.5390 | 0.0501 |  |
| Male last author x Oceania | -6.1308 | -37.6089 | 6.5369 | 0.2036 |  |

**Table B**: Factors predicting whether the manuscript was sent out for review, showing the posterior mode estimates for the fixed effects in the binomial model containing: the gender of the first author (N: male = 1286, female = 973; contrast level = female), the gender of the editor (N: male = 1920, female = 339; contrast level = female), the continent of affiliation (N: Africa = 26, America = 794, Asia = 81, Europe = 1205, Oceania = 153; contrast level = Europe), gender of the last author (N: male = 1693, female = 566; contrast level = female), gender of first author and its interaction with the gender of the editor, year and the manuscript type (N: research paper = 1914, reviews = 88, short notes = 242, special issue = 15; contrast level = research paper). Bold text indicates posterior modes whose 95% credible intervals do not overlap zero, after FDR correction.

|  | Posterior mode estimate | Lower credible interval | Upper credible interval | pMCMC | FDR pMCMC |
| --- | --- | --- | --- | --- | --- |
| Male first author | -0.3513 | -1.1147 | 0.4740 | 0.4109 |  |
| Male editor | -0.2392 | -2.1737 | 1.6544 | 0.8448 |  |
| Africa | 0.0377 | -1.3683 | 1.5247 | 0.9809 |  |
| America | 0.2754 | -0.0836 | 0.6021 | 0.1132 |  |
| Asia | -0.3613 | -1.2681 | 0.5593 | 0.4186 |  |
| Oceania | -0.1351 | -0.7631 | 0.5008 | 0.6654 |  |
| Male last author | -0.2600 | -0.6200 | 0.1012 | 0.1552 |  |
| Male first author x Male editor | 0.1768 | -0.6973 | 1.0327 | 0.701 |  |
| **Year** | **-0.3778** | **-0.6645** | **-0.1466** | **0.0076** |  |
| **Reviews** | **-2.5622** | **-3.7308** | **-1.4100** | **< 6e-04** | **0.003** |
| Short Notes | 0.0849 | -0.4443 | 0.6176 | 0.7570 |  |
| Special Issue | -1.4596 | -3.6771 | 0.6778 | 0.1870 |  |

**Table C**: Factors predicting whether the reviewer agreed to review the manuscript, showing the posterior mode estimates for the fixed effects in the binomial model containing: the gender of the reviewer (N: male = 1509, female = 447; contrast level = female), the gender of the editor (N: male = 1659, female = 323; contrast level = female), the continent of affiliation (N: Africa = 19, America = 683, Asia = 51, Europe = 1066, Oceania = 137; contrast level = Europe), the gender of the last author (N: male = 1463, female = 493; contrast level = female), the gender of the first author (N: male = 1105, female = 851; contrast level = female), the gender of the reviewer and the interaction with the gender of the editor, the gender of the reviewer and the gender of the first author, year and the manuscript type (N: research paper = 1689, reviews = 52, short notes = 200, special issue = 15; contrast level = research paper). Bold text indicates posterior modes whose 95% credible intervals do not overlap zero, after FDR correction.

|  | Posterior mode estimate | Lower credible interval | Upper credible interval | pMCMC | FDR pMCMC |
| --- | --- | --- | --- | --- | --- |
| Male reviewer | -0.1674 | -0.5486 | 0.4454 | 0.7634 |  |
| Male editor | 0.3995 | -0.5436 | 1.2628 | 0.5229 |  |
| Africa | -0.6868 | -1.7479 | 0.6374 | 0.3461 |  |
| America | 0.0381 | -0.2078 | 0.2291 | 0.7875 |  |
| Asia | 0.4639 | -0.2636 | 0.9527 | 0.2150 |  |
| Oceania | 0.1358 | -0.2711 | 0.4972 | 0.6349 |  |
| Male last author | 0.2550 | 0.0633 | 0.5139 | 0.0229 | 0.075 |
| Male first author | -0.0427 | -0.4070 | 0.2848 | 0.6616 |  |
| Male reviewer* Male editor | 0.1029 | -0.4013 | 0.5892 | 0.7048 |  |
| Male reviewer* Male first author | 0.0879 | -0.2520 | 0.5375 | 0.5751 |  |
| Year | 0.1259 | -0.0122 | 0.2296 | 0.0840 |  |
| Reviews | 0.1277 | -0.8410 | 0.9472 | 0.7570 |  |
| Short Notes | 0.0164 | -0.2521 | 0.3919 | 0.7964 |  |
| Special Issue | -0.4219 | -1.9676 | 0.6823 | 0.3690 |  |

**Table D**: Factors predicting the first decision (accept/revise or reject) on the manuscript’s outcome, showing the posterior mode estimates for the fixed effects in the binomial model containing: the gender of the first author (N: male = 1105, female = 851; contrast level = female), the gender of the reviewer (N: male = 1509, female = 447; contrast level = female), the gender of the last author (N: male = 1463, female = 493; contrast level = female), the continent of affiliation (N: Africa = 19, America = 683, Asia = 51, Europe = 1066, Oceania = 137; contrast level = Europe), the interaction between the gender of first author and gender of the reviewer, year and the manuscript type (N: research paper = 1689, reviews = 52, short notes = 200, special issue = 15; contrast level = research paper). Bold text indicates posterior modes whose 95% credible intervals do not overlap zero, after FDR correction.

|  | Posterior mode estimate | Lower credible interval | Upper credible interval | pMCMC | FDR pMCMC |
| --- | --- | --- | --- | --- | --- |
| Male first author | -2.6306 | -9.5350 | 1.5400 | 0.1539 |  |
| Male reviewer | -0.0385 | -1.9630 | 2.0050 | 0.9389 |  |
| Male last author | -1.1725 | -6.7310 | 4.3330 | 0.5267 |  |
| Africa | -15.9858 | -52.2300 | 3.5360 | 0.1031 |  |
| America | 1.8786 | -2.4580 | 7.6350 | 0.4275 |  |
| Asia | 13.1049 | 2.3410 | 29.9600 | 0.0242 | 0.0605 |
| Oceania | -3.5768 | -12.9300 | 4.5070 | 0.4529 |  |
| Male first author x Male reviewer | -0.1094 | -2.5980 | 2.8350 | 0.9288 |  |
| Year | -0.2716 | -3.2220 | 4.6550 | 0.8664 |  |
| **Reviews** | **-11.8153** | **-34.7500** | **-3.7150** | **0.0165** | **0.0206** |
| Short Notes | 2.5859 | -6.0780 | 9.3610 | 0.6323 |  |
| **Special Issue** | **-84.4506** | **-119.0000** | **-37.8100** | **<6e-04** | **0.0003** |

**Table E**: Factors predicting the final decision (accept or reject) on the manuscript’s outcome, showing the posterior mode estimates for the fixed effects in the binomial model containing: the gender of the first author (N: male = 1105, female = 851; contrast level = female), the gender of the reviewer (N: male = 1509, female = 447; contrast level = female), the gender of the last author (N: male = 1463, female = 493; contrast level = female), the continent of affiliation (N: Africa = 19, America = 683, Asia = 51, Europe = 1066, Oceania = 137; contrast level = Europe), the interaction between the gender of first author and gender of the reviewer, year and the manuscript type (N: research paper = 1689, reviews = 52, short notes = 200, special issue = 15; contrast level = research paper). Bold text indicates posterior modes whose 95% credible intervals do not overlap zero, after FDR correction.

|  | Posterior mode estimate | Lower credible interval | Upper credible interval | pMCMC | FDR pMCMC |
| --- | --- | --- | --- | --- | --- |
| Male first author | -1.1688 | -6.1350 | 7.1090 | 0.9135 |  |
| Male reviewer | 0.6212 | -2.3490 | 3.1070 | 0.7481 |  |
| Male last author | -2.8396 | -13.1700 | 1.1210 | 0.1272 |  |
| Africa | -8.8991 | -39.6000 | 25.9400 | 0.6565 |  |
| America | 2.8343 | -1.4340 | 10.7200 | 0.1629 |  |
| **Asia** | **18.0764** | **2.8930** | **52.5500** | **0.0064** | **0.0222** |
| Oceania | -5.5634 | -19.8600 | 4.3560 | 0.1947 |  |
| Male first author x Male reviewer | -0.5357 | -4.6030 | 2.8770 | 0.7150 |  |
| Year | 0.8499 | -3.2450 | 6.0490 | 0.7634 |  |
| **Reviews** | **-16.6072** | **-42.4100** | **-4.1190** | **0.0025** | **0.0041** |
| Short Notes | 2.5114 | -7.2070 | 10.7500 | 0.6756 |  |
| **Special Issue** | **-42.5946** | **-166.000** | **-20.4400** | **< 6e-04** | **0.0003** |
